# ‘European Antibiotics Awareness Day’ wins ‘European Health Award 2016’ accolade

**DOI:** 10.2807/1560-7917.ES.2016.21.39.30354

**Published:** 2016-09-29

**Authors:** Eurosurveillance editorial team

**Affiliations:** 1 European Centre for Disease Prevention and Control (ECDC), Stockholm, Sweden

On 28 September 2016 the ‘European Antibiotic Awareness Day’ (EAAD), a health initiative coordinated by the European Centre for Disease Prevention and Control (ECDC) won the ‘European Health Award’, a prize awarded each year by the European Health Forum Gastein.

The EAAD, one of the six cross-border health projects short-listed for the European Health Award aims to provide a platform to support the prudent use of antibiotics. Each year across Europe, the EAAD is marked by national campaigns during the week of 18 November. The goal of the EAAD is to provide the participating countries with evidence-based tools and other support for their campaigns. In 2015, with the support of World Health Organisation Europe, 41 European countries organised activities to mark the initiative. The EAAD partners with World Antibiotic Awareness Week.

The European Health Award, established in 2007, aims to honour initiatives aiming to improve public health or healthcare in Europe.

Read more about EAAD here

Articles about EAAD in *Eurosurveillance*:

http://www.eurosurveillance.org/ViewArticle.aspx?ArticleId=20928

http://www.eurosurveillance.org/ViewArticle.aspx?ArticleId=19280

